# Incentivising use of structured language in biological descriptions: Author-driven phenotype data and ontology production

**DOI:** 10.3897/BDJ.6.e29616

**Published:** 2018-11-07

**Authors:** Hong Cui, James A. Macklin, Joel Sachs, Anton Reznicek, Julian Starr, Bruce Ford, Lyubomir Penev, Hsin-Liang Chen

**Affiliations:** 1 University of Arizona, TUCSON, United States of America University of Arizona TUCSON United States of America; 2 Agriculture and Agri-Food Canada, Ottawa, Canada Agriculture and Agri-Food Canada Ottawa Canada; 3 University of Michigan, Ann Arbor, United States of America University of Michigan Ann Arbor United States of America; 4 University of Ottawa, Ottawa, Canada University of Ottawa Ottawa Canada; 5 University of Manitoba, Winnipeg, Canada University of Manitoba Winnipeg Canada; 6 Pensoft Publishers & Bulgarian Academy of Sciences, Sofia, Bulgaria Pensoft Publishers & Bulgarian Academy of Sciences Sofia Bulgaria; 7 University of Massachusetts at Boston, Boston, United States of America University of Massachusetts at Boston Boston United States of America

**Keywords:** Controlled Vocabulary, Computable Phenotype Data, Data Quality, Phenotype Ontologies

## Abstract

Phenotypes are used for a multitude of purposes such as defining species, reconstructing phylogenies, diagnosing diseases or improving crop and animal productivity, but most of this phenotypic data is published in free-text narratives that are not computable. This means that the complex relationship between the genome, the environment and phenotypes is largely inaccessible to analysis and important questions related to the evolution of organisms, their diseases or their response to climate change cannot be fully addressed. It takes great effort to manually convert free-text narratives to a computable format before they can be used in large-scale analyses. We argue that this manual curation approach is not a sustainable solution to produce computable phenotypic data for three reasons: 1) it does not scale to all of biodiversity; 2) it does not stop the publication of free-text phenotypes that will continue to need manual curation in the future and, most importantly, 3) It does not solve the problem of inter-curator variation (curators interpret/convert a phenotype differently from each other). Our empirical studies have shown that inter-curator variation is as high as 40% even within a single project. With this level of variation, it is difficult to imagine that data integrated from multiple curation projects can be of high quality. The key causes of this variation have been identified as semantic vagueness in original phenotype descriptions and difficulties in using standardised vocabularies (ontologies). We argue that the authors describing phenotypes are the key to the solution. Given the right tools and appropriate attribution, the authors should be in charge of developing a project’s semantics and ontology. This will speed up ontology development and improve the semantic clarity of phenotype descriptions from the moment of publication. A proof of concept project on this idea was funded by NSF ABI in July 2017. We seek readers input or critique of the proposed approaches to help achieve community-based computable phenotype data production in the near future. Results from this project will be accessible through https://biosemantics.github.io/author-driven-production.

## Introduction

Phenotypes are paramount for describing species, studying function and understanding organismal evolution. Recent advancements in computation technology have enabled large-scale, data-driven research, but its full potential has not been realised due to lack of data. High impact research, such as studying trait evolution and its relationship to phylogeny and the environment (e.g. Zanne et al. 2013; Pender 2016), identifying candidate causal genes based on known genotype-phenotype relationships in other taxa (e.g. Edmunds et al. 2015) and resolving taxon names through analysing the relationships between taxonomic concepts with character-based evidence (e.g. Franz et al. 2015; Cui et al. 2016) cannot be realised at this scale without computable phenotype data being available for every clade and taxonomic group.

Textual phenotype descriptions that hold valuable information are continuously being published, yet they are not amenable to computation. When added to the massive amount of phenotype data sitting in older publications, these free-text character descriptions represent a major, under-utilised resource for integrating phenotypic data into modern, large-scale biological research projects that typically involve genomic, climatic and habitat data. These descriptive data are often variable in expression and terminology. Different descriptions of the same character may appear to describe two different traits or two different characters might be interpreted as one. Transforming various natural language expressions into computable data requires a process, called ontologising, where the semantics (meaning) of varied expressions are mapped to terms in an ontology and therefore made explicit (Mabee et al. 2007). An ontology holds a set of well-defined terms and their relationships, for example, *leaf* and *petiole*, have a relationship: all *petioles* are *part of* some *leaf*. Ontologising ensures “apples are compared to apples” and forms the foundation for meaningful data integration and machine inference and reasoning (i.e. inferring new facts from given facts). For example, if *leafstalk* is equivalent to *petiole*, then all *leafstalks* are *part of* some *leaf* as well.

Currently, making free-text phenotype information computable requires highly trained post-doctoral researchers manually ontologising the descriptions, facilitated by some software applications. However, the manual curation of legacy descriptions is not a sustainable solution for phenotype data production because it does not stop the continued publication of free-text phenotype descriptions that need semantic curation before use. If we assume that each of the estimated 750,000 biomedical papers published in English in 2014 (Ware and Mabe 2015) mentions just one phenotypic character and each character takes about 5 minutes to curate (Dahdul et al. 2015, personal communication with Dahdual), one year’s worth of English biomedical journal publications alone would take a full-time postdoc over 30 years to curate.

Manual curation also does not address the fundamental causes of large (~ 40%) variations in the phenotype data manually curated by different workers (e.g. Cui et al. 2015; Manda et al. in press). This level of variation is concerning because ontologised characters must be highly accurate for computers to produce sound inferences or support data integration. In detailed analyses, two major underlying causes of variation were revealed: incomplete, hard-to-use ontologies and semantic ambiguities in source descriptions (Cui et al. 2015; Huang et al. 2015). Neither of these problems can be adequately addressed by manual curation or text-mining techniques because computers are at their weakest with semantic and pragmatic analyses and cannot be expected to perform better than highly trained humans.

As long as phenotype descriptions continue to be produced as free text, computable phenotype data will remain a major bottleneck holding back large-scale biological research. Given the varied usages of phenotype terms/expressions by different authors and given the fact that the meanings of a term evolve over time, it is evident the semantics of phenotypic characters (categorical or continuous characters) can be most accurately captured at the time of writing by their authors. Any downstream process risks information loss or even misinformation.

## Author-Driven Phenotype Data and Ontology Production

We have been awarded funding to investigate a new paradigm of phenotype data production centred on description authors and supported by intuitive software tools to allow them to compose semantically clear descriptions while contributing their vocabularies/expressions to a shared ontology for their taxon groups. It brings authors to the forefront of ontology construction, promotes clear expressions and exposure of all valid meanings of technical terms and encourages open collaboration and consensus building amongst scientists. While the proposed approach presents a major conceptual change in phenotype data authoring, the change can be introduced via software environments with which authors are already familiar, for example, Google Docs and Wikis. We will approach the project from the perspectives of social and software engineering, examining human social and collaborative behaviour (e.g. attribution and motivation) and software usability to identify factors that encourage or discourage users from adopting the approach. Although we will start with a test case using the plant genus *Carex* L. (“sedges”, family Cyperaceae), the project has the potential to change how biodiversity is described in general and dramatically ease the production of computable phenotype data at a large scale.

Using the ongoing *Carex* revisionary work as the evaluation case for this project is an excellent choice because: 1). *Carex* is one of the largest genera in flowering plants, with close to 2000 species containing considerable variation. 2). A network of *Carex* experts already work closely to prepare the revisions. 3). *Carex* is treated in Flora of North America and Flora of China, from which we have previously extracted over 1200 *Carex* morphological terms and will be used to build the initial *Carex* Phenotype Ontology (CPO) for scientists to improve and 4). Scientists on this project will use the large amount of characters produced from this approach to expand their past research to a scale not possible before (Pender 2016). We are not advocating the creation of more ontologies as randomly creating ontologies will only create new challenges for the end users. What we are arguing is that any phenotypic ontologies created must be *directly* useful to the scientists. If some of these usages are out of the scope of existing ontologies (e.g. in the case of plants, the Planteome Consortium Ontologies), they need to be addressed by more specific domain ontologies, in consultation with the exisiting ontologies. In the Carex case, the Author's project and the Planteome project have made a clear roadmap in terms of when to reuse terms and relations from the Plant Ontology and when to create new terms for the Carex Ontology. We feel that getting the buy-in at this time from the authors is the most critical mission, while developing successful ontology development strategies, a valuable side product, is of a secondary concern, at least for this project.

We also note that the larger academic and scientific research environment support the premises of the proposed approach. The importance of computable phenotype data is widely recognised and data silos are being actively dissolved. Ontologies and other data publications are valued and attribution methods are being actively examined to credit intellectual contributions to digital resource curation, such as the efforts by the International Society for Curation (http://biocuration.org), OBO Foundry (http://www.obofoundry.org) and THOR (http://project-thor.eu). Publishers like Pensoft are actively seeking and welcoming new methods to stop the continued publication of legacy descriptions. Having years of experience with using digital tools/devices, scientists are expert users of digital collaborative environments (e.g. Wikis, Google Docs). The time is right to investigate a long-term solution to phenotype data production.

## Proposed System Design

**Figure 1.**
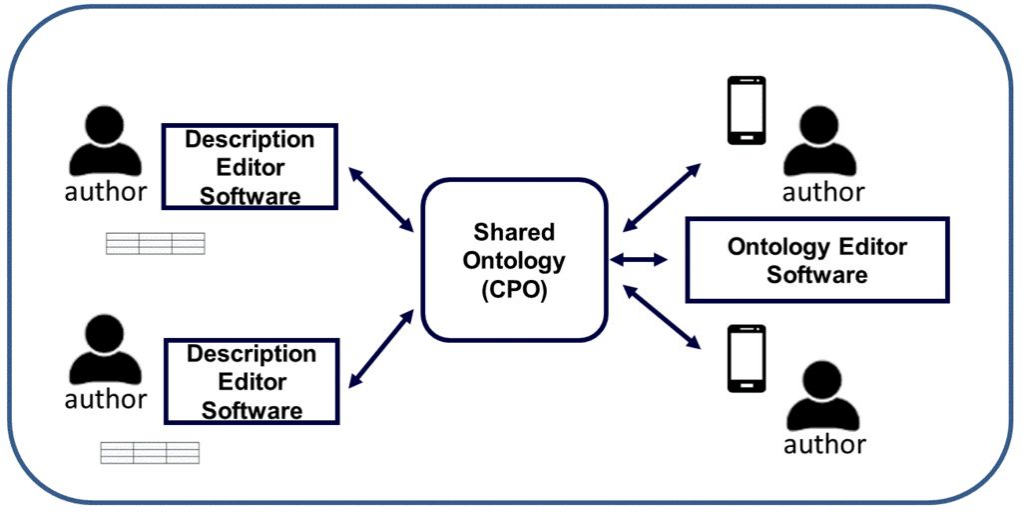
The Integrated Description and Open Collaborative Ontology Editing Platform, with taxon-character matrices by-products. Notice that description authors are also ontology authors.

Fig. 1 illustrates the prototype that will be developed and evaluated in the project. Analogous to Google Doc or Microsoft Word Editor’s Spell Checker and personal dictionary, a semantic-aware Description Editor can be used to check semantics using a shared ontology. By making it easy for all authors to add their term usages to a shared ontology and to relate new terms to existing terms in the ontology, a taxon-specific phenotype ontology then comprehensively covers the terms and relationships of the descriptors (i.e. the domain) used by the author community. By revealing how terms are used within a community setting, authors are encouraged to converge to best practices in describing certain characters. This process offers two key benefits: (1) the authors are free to use their terms of choice and (2) author terms are related explicitly to other terms in the ontology so the meaning of the terms is clear. This results in descriptions with clear semantics, making ontologisation of the characters a straightforward step for composing formal statements by harvesting the semantics already expressed in the descriptions. This is then a task that computers can do more efficiently than curators. In addition, the community will quickly have a comprehensive ontology that is tested by use.

It is important to differentiate this approach from a standardisation approach where the authors are limited to using a set of “standardised” terms selected by others. The proposed approach does not limit author's choices, but it requires the authors to register the meaning (i.e. semantics) of the terms in their descriptions in an ontology and relate them to other existing terms to allow accurate interpretations in the future. For example, a standardisation approach might require Joe to use the term *strong* when he wishes to say *stout*. In contrast, our approach might show Joe that *stout* has two related but different meanings: *increased size* and *strong (not fragile)*. This would allow Joe to choose the most precise term to use, *increased size*, *strong* or *stout* and, in turn, allow the reader, human or computer, to obtain the accurate meaning intended by the author. The key idea of the proposed approach is to make all valid meanings of a term clear and visible to a community of users and to encourage the user to filter and choose terms with the most accurate meaning for their purposes.

**Figure 2.**
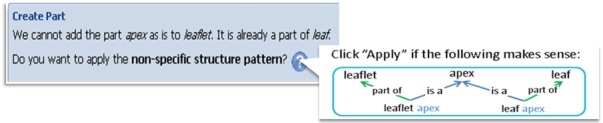
The system detects that the user is attempting to add a substructure (apex) to multiple parent structures (leaf and leaflet). This triggers the system to suggest the non-specific structure pattern to the user. When the user confirms, the system will insert four assertions (4 links in the graph) into the ontology automatically.

When the user adds a term to the ontology, the online open Ontology Editor is invoked, presenting different patterns to relate the terms in semantic ways (e.g. assert *utricle in Carex ≡ perigynium in Carex, spike is_a inflorescence, spikelet ≡ secondary spike or small spike, stout* ≡*strong and increased size, weak* ≡*decreased magnitude or decreased strength*). Ontology design patterns (e.g.Egaña et al. 2008; Presutti et al. 2012) can be used to wrap the complexity of the logic in a friendly user interface so that users lacking description logic training can use them. For example, non-specific structures, such as apex, surface and base, that can be part of many different structures need to be treated with several logic assertions. Our software can detect cases like this and automatically generate the complete set of assertions for the user to approve (Fig. 2).

These patterns are expected to greatly improve the predictability of the ontology, reduce variation and lower the barrier to entry for biologists. The software will auto-detect situations, whenever possible, for which a pattern may be useful; once the user confirms, the system will carry out what needs to be done on the user’s behalf. Fig. 2 illustrates such a scenario for the non-specific structure pattern described above.

Small ontology building tasks such as conflicts amongst term definitions and relationships can be broadcast via a simple mobile app for registered authors to resolve at their leisure. Technical challenging cases can be resolved with help from trained ontology engineers, for example, the Planteome Project (http://planteome.org) or the OBO Foundry.

The rewards to authors who adopt this new workflow include: (1) Narrative descriptions in camera-ready form for publication. (2) A taxon-by-character matrix formulated ontology terms, ready for publication. These can be published in partner journals (e.g.Pensoft journals) in a customisable human readable form (e.g. sentences or matrices) and a variety of new ontologised formats such as EQs (Entity Quality) in the Phenoscape Knowledgebase (http://kb.phenoscape.org) or RDF graphs (Resource Description Framework, a format used widely on the Semantic Web). (3) Formal attributions and increased citations. On one hand, research has shown that studies that make their data available receive more citations than similar studies that do not (e.g. Piwowar and Vision 2013) and, on the other hand, terms added to the ontology can be linked to the Open Researcher and Contributor ID (ORCID) and the name of the authors and packaged as a micro-publication with a DOI. This could give data consumers another way to include formal data citations in their publications. Even though current data citation practices vary (Robinson-Garcia et al. 2016), the trend is clear as the support for data citations has been widely seen cross disciplines, from science (e.g. Gupta et al. 2017, Cook et al. 2016) to social sciences (e.g. Berez-Kroeker et al. 2017) and from libraries (e.g.Brase et al. 2015) to publishers (e.g.Pavlech 2016). (4) Achievement badges earned based on their contributions within the platform and visible to colleagues.

Results from social and behavioural sciences research on computer mediated collaborative work, online community building and consensus making (e.g. Grudin 1988;Grudin 1994; Innes and Booher 1999; Kriplean et al. 2007; Krieger et al. 2009; Choi et al. 2010; Halfaker et al. 2014; Janssen et al. 2014; Mason et al. 2016) will be implemented to guide the user interaction design of the above-described prototype platform. We acknowledge and have personally witnessed the fact that user participation in open collaborations is often uneven (Wilkinson 2008), but we will strive to design a system where users with different motivations, skill sets and preferences can be engaged in activities that contribute to the overall goal (Preece and Shneiderman 2009;Lampe et al. 2010; Panciera et al. 2010; Wohn et al. 2012; Morgan et al. 2014). While investigating ways to build a strong core of contributors and leaders (Zhu et al. 2012; Luther et al. 2013), steps and design lessons can be taken to integrate and retain new users (Choi et al. 2010; Halfaker et al. 2011; Halfaker et al. 2014; Steinmacher et al. 2015). This project continues our quest to build low barrier software for biologists based on the existing knowledge of what works to encourage open collaboration and consensus making and also contribute to an understanding of the scientific consensus-making process via the new botanical research we plan to conduct with our tools.

## Expected Results

We hypothesise that, with careful design of the user interface that takes into account user-friendliness, efficiency, user motivation and other social and behavioural factors, this approach will increase phenotype data quality, ontology quality and computation efficiency.

1. Data quality: improve the semantic clarity of new phenotype descriptions to dramatically reduce the scope of the subsequent ontologisation effort,
2. Ontology quality: quickly improve the coverage of the phenotype ontology for a particular domain (e.g. a taxonomic group) and
3. Computation efficiency: obtain ontologised matrices and/or EQ statements with higher consistency and hence support a wide range of applications.

Assuming this proof of concept system is successful, this approach can be applied to any other science and engineering domains (e.g. biomedical, geology, astrophysics etc.). This being so, individual domain ontologies can be linked, based on shared concepts and terms, thus building powerful bridges for integration across domains, sciences and beyond.

## Conclusion

Readers interested in learning more about our project and eventually evaluating our software prototypes can obtain further information from our github project page (https://biosemantics.github.io/author-driven-production) or contact authors. In summary, the goal of this project is to investigate the feasibility of transforming phenotype authors’ writing practice to produce computable phenotype data at the time of publication, with increased speed, scale, quality and consistency, while collectively curating phenotype ontologies, making them reflect a community consensus. Through thorough user experience research, we will also identify ways to reduce the entry barrier and promote user adoption of the new practice. When publishers adopt this new idea, we believe the ultimate goal of producing massive high-quality phenotype data for the entire scientific community can be achieved. We seek readers input or critique of the proposed approaches to help achieve community-based computable phenotype data production in the near future.

## Hosting institution

University of Arizona
